# Trait anxiety modulates the detection sensitivity of negative affect in speech: an online pilot study

**DOI:** 10.3389/fnbeh.2023.1240043

**Published:** 2023-09-07

**Authors:** Achyuthanand K, Saurabh Prasad, Mrinmoy Chakrabarty

**Affiliations:** ^1^Department of Computational Biology, Indraprastha Institute of Information Technology Delhi, New Delhi, India; ^2^Department of Computer Science and Engineering, Indraprastha Institute of Information Technology Delhi, New Delhi, India; ^3^Department of Social Sciences and Humanities, Indraprastha Institute of Information Technology Delhi, New Delhi, India; ^4^Centre for Design and New Media, Indraprastha Institute of Information Technology Delhi, New Delhi, India

**Keywords:** acoustic emotion, anxiety, disgust, fear, signal detection theory

## Abstract

Acoustic perception of emotions in speech is relevant for humans to navigate the social environment optimally. While sensory perception is known to be influenced by ambient noise, and bodily internal states (e.g., emotional arousal and anxiety), their relationship to human auditory perception is relatively less understood. In a supervised, online pilot experiment sans the artificially controlled laboratory environment, we asked if the detection sensitivity of emotions conveyed by human speech-in-noise (acoustic signals) varies between individuals with relatively lower and higher levels of subclinical trait-anxiety, respectively. In a task, participants (*n* = 28) accurately discriminated the target emotion conveyed by the temporally unpredictable acoustic signals (signal to noise ratio = 10 dB), which were manipulated at four levels (Happy, Neutral, Fear, and Disgust). We calculated the empirical area under the curve (a measure of acoustic signal detection sensitivity) based on signal detection theory to answer our questions. A subset of individuals with High trait-anxiety relative to Low in the above sample showed significantly lower detection sensitivities to acoustic signals of negative emotions – Disgust and Fear and significantly lower detection sensitivities to acoustic signals when averaged across all emotions. The results from this pilot study with a small but statistically relevant sample size suggest that trait-anxiety levels influence the overall acoustic detection of speech-in-noise, especially those conveying threatening/negative affect. The findings are relevant for future research on acoustic perception anomalies underlying affective traits and disorders.

## Introduction

Appraisal of emotions pervades everyday human social interactions in various ways. Correct recognition of emotional signals through different sensory modalities, e.g., vision, voice, and touch, influence a nuanced understanding of the external social environment with graded overtones (negative to positive), enabling the human brain to generate behavior that is aptly calibrated to meet the demands of the complex social situation. Accurate discrimination of emotions inherent in social signals, therefore, is critical to deduce the expresser’s reactions to preceding events and present state of mind and predict possible future actions. This enables one to engage in reciprocal interpersonal interactions and adjustments in efficiently navigating the social landscape (Scherer, 2009; Ong et al., 2015). While several studies have explored the role of emotions conveyed by visual stimuli (e.g., human faces) on various cognitive functions (Phelps et al., 2006; Chakrabarty and Wada, 2020; Chakrabarty et al., 2021; Kaur et al., 2023), reports in this domain of the auditory modality (e.g., human voices) are relatively sparse (Schirmer and Adolphs, 2017). Taking account of this lacuna is relevant since voices are an essential modality for communicating emotions in our daily environments. Voices are generated by the complex functioning of the different organs of the vocal system signaling innate and learnt emotions, and in turn, could be influenced by the emotion-induced physiological changes such as breathing and muscle tone of the vocal system (Schirmer and Adolphs, 2017). So, voices convey important emotional information through the prosodic parameters (e.g., pitch, duration, semantic contents), which can be differentiated from verbal content (Cummings and Clements, 1995; Banse and Scherer, 1996). Acoustic speech perception occurs through several steps in the brain, involving a cross-talk between bottom-up sensory-driven and top-down experience-dependent processes (Schroeder et al., 2010). Through these processes, humans are known to perceive the emotions of even unfamiliar speakers through perturbations or deviations of their typical state of speech, such as an increase in pitch, intensity, or tempo (Bachorowski, 1999; Paidi and Kadiri, 2016).

Incidentally, the detection of sensory signals for higher-level cognitive functions is also influenced by the internal states of the body, e.g., arousal and anxiety, owing to the bidirectional autonomic nervous system projections across the prefrontal, limbic and sensory networks of the brain regulating the processing of sensory inputs (Aston-Jones and Cohen, 2005; Critchley, 2009; Critchley and Harrison, 2013). Several studies examining visual functions in humans corroborate the above, e.g., contrast and orientation sensitivity, temporal resolution, and attentional topography, to list just a few (Ferneyhough et al., 2013; Barbot and Carrasco, 2018; Chakrabarty et al., 2021; Kanae et al., 2021; Kaur et al., 2023). Similar biases of auditory functions by the individual’s internal state have also been reported in animals and humans alike. For example, it has been shown that mice trained to perform a tone-in-noise signal detection task perform the best (in terms of speed, accuracy, and magnitude of responses) with an intermediate state of arousal that corresponded with a high signal-to-noise ratio of evoked cortical responses from the brains of mice (McGinley et al., 2015). Several studies in humans also suggest that internal states of anxiety and arousal influence auditory sensory functions behaviorally. For example, high compared to low socially anxious individuals had lower accuracy and longer reaction times with angry (negative) relative to neutral when attending to emotional prosody in a dichotic listening task (Peschard et al., 2017). This finding in individuals with high social anxiety is substantiated by another study that found lower accuracy and longer reaction times in recognizing fearful prosody (Tseng et al., 2017) as well as greater misattribution of anger (negative) to otherwise neutral vocal emotions (Peschard and Philippot, 2017). In line, the generalized social phobia has been reported to bias the processing of emotional prosody in a pilot study. However, individuals with phobia showed enhanced identification of sad and fearful (negative) and decreased identification of happy voices compared to healthy controls (Quadflieg et al., 2007).

Further, using a signal-in-white-noise detection task, a study reported that adult individuals with high as compared to low anxiety sensitivity had marked lower ability (characterized by increased false alarm rates) to discriminate sounds of abnormal heartbeats from background noise as well as between abnormal and normal heartbeats (Pollock et al., 2006). A related pattern of acoustic perception has been reported even in children with high anxiety sensitivity (Pollock-Wurman et al., 2011). Apart from recognition accuracies of emotions, a negative interpretation bias of vocal emotions from face-voice pairs has also been reported in high-trait anxious individuals (Koizumi et al., 2011). On the other hand, studies manipulating arousal by inducing emotional states have reported that not only do brain steady-state responses evoked by auditory stimuli vary significantly between induced states of different emotional valences (Zhang et al., 2021), but they also influence acoustic perception, thereby serving as a basis for categorizing everyday sounds (Bergman et al., 2016).

Anxiety is a mental state characterized by intense apprehension of uncertain future adverse event(s) without any transparent object, and excessive levels can be dysfunctional, leading to anxiety disorders (Grillon, 2008). Healthy individuals may not meet the clinical threshold for categorical diagnosis of anxiety disorders but still have varying degrees of behavioral predispositions to judge otherwise innocuous events as potentially threatening, e.g., in subclinical state- and trait-anxiety (Spielberger, 1983), with those on the higher levels of the trait-anxiety spectrum at future risk of developing clinical anxiety and other affective disorders (Weger and Sandi, 2018). Trait-anxiety is linked with emotions in biasing cognitive functions, such as visual attention (Phelps et al., 2006; Ferneyhough et al., 2013; Barbot and Carrasco, 2018; Kaur et al., 2023) and interpretation of vocal emotions (Koizumi et al., 2011). Here, earlier studies suggest that while there are attentional biases towards negative stimuli of threatening potential among anxious population, attentional biases to stimuli may also be a result of the recruitment of fear circuits as a defensive response in individuals with anxiety or an anxious predisposition. Additionally, studies have shown that learned attentional biases towards stimuli can also lead to anxiety (Cisler et al., 2009, 2010). Thus, anxiety may not only facilitate the detection of negative emotional stimuli (Schirmer and Escoffier, 2010; White et al., 2010; Berggren et al., 2015; Barbot and Carrasco, 2018) but also worsen the same (Montagne et al., 2006; Demenescu et al., 2010) and a clear line of causation remains to be established. However, whether trait-anxiety levels in otherwise healthy individuals also influence the acoustic perceptual classification accuracy of emotions against background noise, is less understood, to our knowledge. We thought this direction of enquiry plausible given the association between anxiety, emotions, and sensory encoding of stimuli in the biological neural networks (Vuilleumier, 2005; Bishop, 2007; David, 2018). Besides, all the above studies have been conducted with experimenter-controlled ambient sound levels in the laboratory environment, and it remains to be seen whether the interaction between trait – anxiety, and emotions additionally influences the accuracy of detecting emotional valences from a noisy speech in less controlled ambient backgrounds.

To the above end, we presented four vocal emotions (Happy-HA, Neutral-NE, Fear-FE, and Disgust-DI) inherent in an otherwise semantically neutral human speech against background white noise (speech-in-noise) to human participants. We tested whether the average acoustic perceptual emotion detection accuracies varied between subsets of participants with relatively higher (High Anxiety) and lower (Low Anxiety) severities of trait-anxiety within the sample. The speech stimuli were chosen to represent the emotional dimensions of affective valence (pleasure-displeasure continuum) as well as arousal (high-low continuum), i.e., negative × arousing (FE and DI); positive × arousing (HA) and neutral in both dimensions (NE) as per the models of affect (Russell, 2003; Posner et al., 2005). It is pertinent to mention here that emotions also have a motivational dimension independent of affective valence, i.e., approach vs. withdrawal relative to the elicitor (Harmon-Jones, 2019). In this study, we focussed on emotions that have a motivation of approach (HA), withdrawal (FE and DI), and neutral (NE). It is also worth mentioning here that the DI emotion was especially chosen because it is one of the basic human emotions with a wide range of elicitors. Its expression is highly variable and believed to have evolutionary and social cognitive implications. Moreover, it has been proposed to be associated with anxiety disorders by synergizing with the FE emotion (Woody and Teachman, 2000; Chapman and Anderson, 2012; Rottman et al., 2019). We found a significantly lower classification accuracy, specifically of FE and DI emotions from human speech-in-noise in the High Anxiety subset as compared to the Low Anxiety subset. The overall classification accuracy across all emotions also followed the same trend. Our findings add to the earlier evidence that inference of emotions from human speech is awry in higher levels of subclinical trait-anxiety.

## Methodology

### Participants

We recruited 28 participants (mean ± standard deviation, SD: age = 23.52 ± 1.75 years, 15 Females,13 males) who did not report any neurological and/or psychiatric diagnoses at the time of the experiment and were naive to experimental goals, after informed written consent to participate in the experiment for a suitable remuneration. All participants were college / university students (after completion of senior secondary education) at the time of their study participation and were adequately versed in English (written and oral) to understand the items of the self-reported questionnaire(s) and meet the overall study requirements.

The present study being a pilot was carried out with lesser resources to gather preliminary data to assess the feasibility of answering our specific questions in future larger-scale, online studies without the strict in-lab control of the experimental environment. Towards this end, an appropriate sample size for this pilot study was reached as follows. From relatively few studies of auditory emotion recognition in non-clinical (healthy) younger adults, a recent study reported (Simonetti et al., 2022) a significant main effect of different emotion valences on auditory emotion recognition accuracies (analysis of variance, effect size: partial *η^2^* = 0.40). The partial *η^2^* value, when converted to Cohen’s *f*, resulted in an effect size of 0.44 which, when entered with a two-tailed alpha level = 0.05 and power of detection = 0.8 into *a priori* sample size calculation for within-between factors interaction for ‘F tests in the G*power version 3.1.9.7 (Faul et al., 2009), yielded at least 10 participants per independent group (Low and High trait-anxiety; see Results for median split) to correctly reject the null hypotheses with 80% chance. This sample size generally agrees with other recommendations for samples in pilot studies of psychological and biomedical measurements (Robin, 1998; Julious, 2005; Hertzog, 2008; van Belle, 2008). Please note that since this was a pilot study, we did not pre-screen the participants based on STAI scores for recruiting them into Low vs. High trait-anxiety groups and also see the Discussion for future considerations in this regard.

Recruitment was done through email advertisement, and participant task performance was assessed in an experimenter-supervised, internet-based (online) experiment (see Experimental Procedures). All test participants were screened for normal hearing acuity from their performance on an online audiometric evaluation test which estimates an individual’s hearing ability to distinguish words and numbers in a noisy environment (ReSound, 2021). In a remotely conducted online experiment, ensuring the quality of headphones is essential to control the presentation and manipulation of acoustic/auditory stimuli to the participants. Thus, the participants’ headphone quality was remotely screened next using two online psychophysical tests – one based on antiphase tones (Woods et al., 2017) and another based on Huggins Pitch (HP), a perceptual phenomenon only detected when auditory stimuli are presented dichotically (Milne et al., 2021). It has earlier been demonstrated that pairing the above headphone screening tests markedly lowers the false positive rates of auditory detection (Milne et al., 2021). The study and its procedures were approved by the Institutional Ethics/Review Board of the Indraprastha Institute of Information Technology Delhi, INDIA.

### Subjective ratings

All participants completed the state–trait anxiety Inventory, STAI (Spielberger, 1983) for assessment of self-reported intensities of anxiety. Trait-anxiety is a relatively stable disposition of an individual to judge a range of otherwise innocuous events as potentially threatening and an indicator of the likelihood of the person responding to perceived threats over time (Wiedemann, 2015; Spielberger, 2022). The trait-anxiety part of the STAI has 20 items with response options from ‘not so much’ to ‘very much so.’ Its total score ranges from 20–80, with higher scores indicating more significant anxiety. Test–retest coefficients have ranged between 0.69 to 0.89 over a two-month interval (Spielberger, 1983). The questionnaire was administered online after the participants passed the hearing acuity and headphone screening test but before beginning the practice trials of the actual experiment.

### Experimental procedures

**Figure 1:**
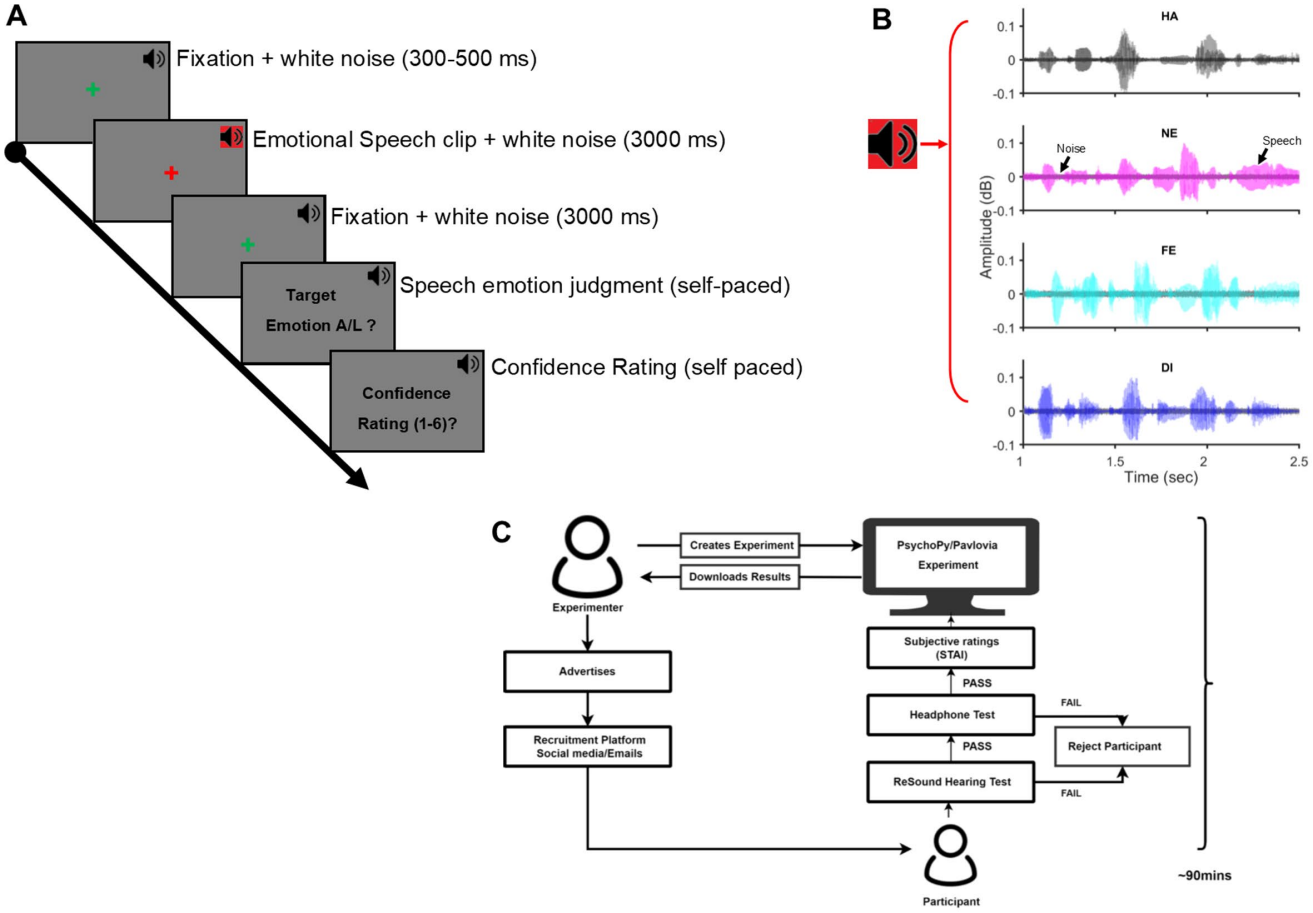
Experimental procedures: **(A)** The timeline of one trial of the behavioral task is shown. A green fixation cross at the screen centre for the pseudorandom duration (300–500 ms) was immediately followed by an emotional speech clip embedded in white noise for 3,000 ms (SNR = 10 dB). Following a fixation cross for 3,000 ms, a speech emotion judgment task and, next, a subjective confidence rating task of the prior judgment was presented in a self-paced duration. The next trial started after a blank of 500 ms. Throughout the trial, a background white noise (37 dB; black speaker icon) was presented. **(B)** Time waveform of Speech signal embedded in Gaussian white noise for Happy (HA), Neutral (NE), Fear (FE), and Disgust (DI) emotion. **(C)** Illustration of the experiment workflow, beginning with the advertisement for participant recruitment, passing the hearing acuity and headphone tests to the conclusion of the main experiment. Each participant spent an average of 90 min in the experiment session; SNR, signal-to-noise ratio.

Participants took the entire experiment over the internet remotely from their accommodations. The entire process was supervised and remotely controlled by an experimenter to ensure that the participants were in a relatively quiet environment of their accommodations during the study duration and adhered to all task demands of the experiment. Participants entered a two-way video call platform on a personal computer (PC) with a functional headphone and completed the entire experiment in one sitting but with intermittent breaks between the task sessions. Each participant spent, on average, 90 min for the entire experiment. The experiment was controlled by custom code written in PsychoPy v2021.2.1 (Peirce, 2009) and hosted on Pavlovia (Bridges et al., 2020), a web-based platform to host and conduct experiments remotely. After passing the hearing acuity and headphone tests, participants were first acquainted with the main experiment with 10–20 practice trials wherein the speech stimuli were different from those used during the actual experiment. The main experiment proceeded after ensuring the participants’ complete understanding of the task demands. Each trial of the main experiment began with a fixation cross at the centre of the computer screen, presented randomly for 300–500 ms. This was followed by the speech signal simultaneously masked by a background white noise signal (speech-in-noise). The speech signal was simultaneously masked by a background white noise signal (speech-in-noise acoustic signal; signal-to-noise ratio = 10 dB, average duration = 3,000 ms) followed. After an equal intervening blank of 3,000 ms coinciding with a fixation cross at the screen centre, an instruction screen signaled participants to discriminate the speech-in-noise into two categories and after that, indicate their confidence in the preceding judgment by pressing appropriate keys on their personal computer keypad. No time restriction was imposed for the two keypress responses. The trial ended with keypress and the next trial began after a blank of 500 ms. Participants were explicitly instructed to maintain fixation at the screen centre throughout the trial, stay alert to listen to the acoustic signal, accurately discriminate the category of the acoustic signal (target / non-target) as well as assess their subjective confidence (scale:1 [least] – 6 [most]) in the preceding judgment and to finally indicate their decisions in the last two steps accurately and speedily by an appropriate key press. Figure 1 illustrates the experimental design.

Each participant performed four sessions (one session each with four different Target acoustic emotion signals: Happy, Neutral, Fear, and Disgust) of 60 trials each, with intermittent breaks (total = 240 trials). One session consisted of 30 trials each of target and non-target acoustic emotion signals (total = 60 trials) presented randomly. The non-target acoustic emotion signals in each session were drawn from the three emotion categories (10 signals each) other than the target emotion for that session. The target emotion to be identified in a given session was informed to the participants on their PC screens at the start of the session, and the order of presentation of target emotion sessions was randomized across participants. The central hypothesis was that the sensitivity of detecting emotional valences conveyed by acoustic signals, i.e., semantically neutral human speech embedded in background white noise differs between the two subsets of the participants with High and Low trait anxiety.

The speech stimuli (conveying HA, NE, FE, and DI target emotion) were sourced from The Ryerson Audio-Visual Database of Emotional Speech and Song (RAVDESS), comprising speech files from 24 actors. They are neutral sentences, e.g.,” Kids are talking by the door,” spoken in different emotional intonations, and have reported good emotional validity, test–retest and intra-rater reliability (Livingstone and Russo, 2018). Thirty speech stimuli of each target emotion (15 female and male actors, respectively) and normal intensity were used. Thus, in any given target emotion session, the ten non-target emotional speech stimuli of each of the other three categories comprised five female and five male stimuli, making a total of 30 non-target stimuli. Any given speech stimulus was presented once in a session and four times during the experiment. The speech stimuli were processed by the following steps before presenting them to the participants. Gaussian white noise was added to each speech stimulus after measuring the power of the speech stimulus such that the signal-to-noise ratio (SNR) was 10 dB as in simultaneous masking and was done using the ‘awgn’ function in the Communications Toolbox of MATLAB 2020b. Each noise-masked speech stimulus (acoustic signal) was then trimmed to the first 3,000 ms without excluding speech information and then used for the experiment.

### Data analysis

Data from the keypad responses were analyzed by custom codes, and the following explains the analysis for one participant. Within one session, the response data were first sorted by each of the four Target emotions (HA, NE, FE, DI). The trials wherein “Target emotion” in acoustic signals were reported given that the acoustic signals were the target (Signal Trials) were defined as “Hits.” By contrast, the trials wherein ‘target emotion’ in acoustic signals were reported given that the acoustic signals were not the target (Noise Trials) were defined as “False alarms.” The number of “Hits” and “False alarms” together with the total number of Signal and Noise trials were entered into the following equations to calculate two metrics of signal detection theory (SDT) as explained elsewhere (Stanislaw and Todorov, 1999), i.e., Hit Rate (Proportion Hits) and the False alarm Rate (Proportion False alarms).


(1) $$\frac{\left(Hits+0.5\right)}{\left(Signal\;trials+1\right)}$$

(2) $$\frac{\left(False\;alarms+0.5\right)}{\left(Noise\;trials+1\right)}$$

**Figure 2:**
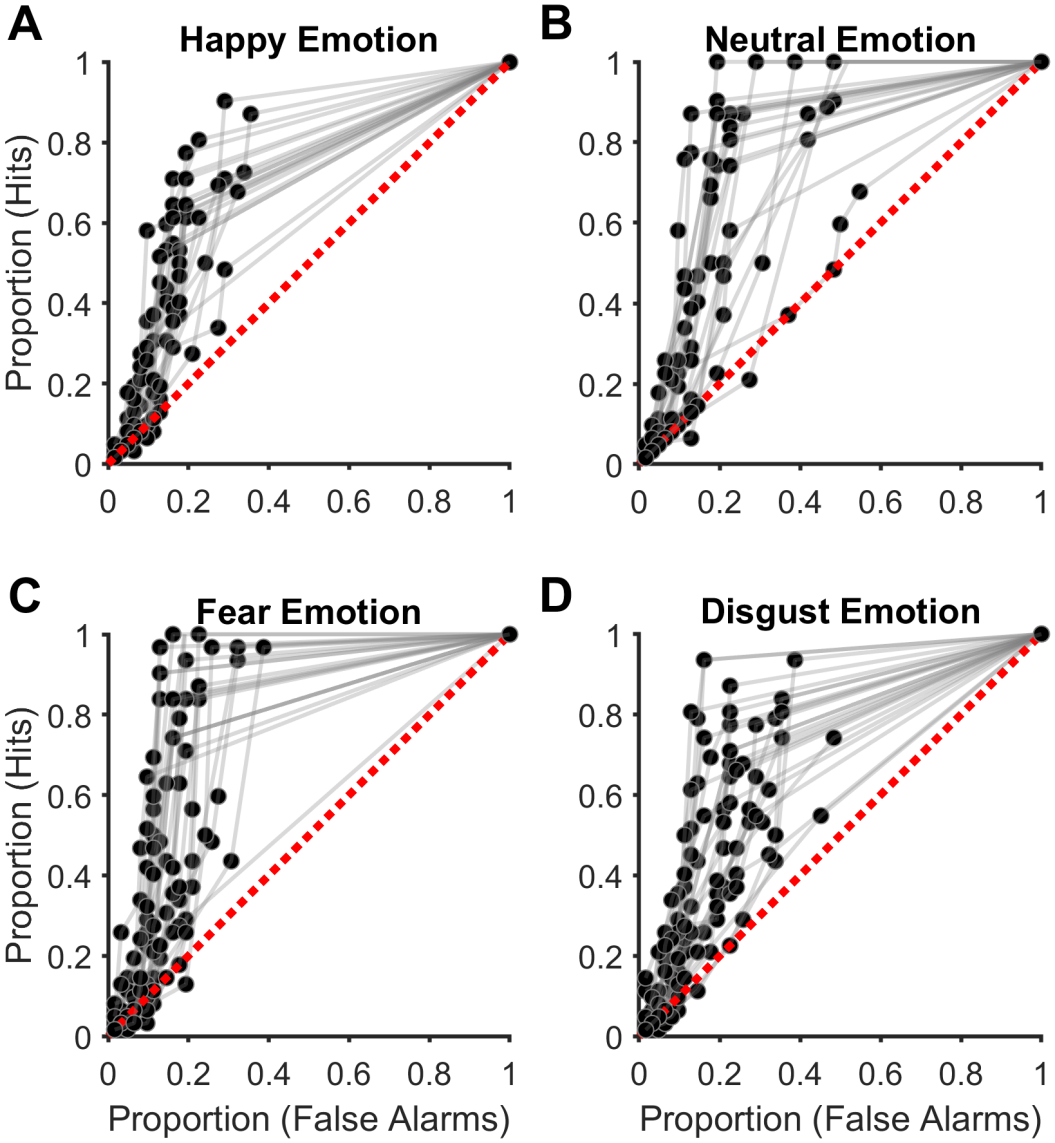
Discrimination accuracy of acoustic emotions. **(A–D)** Plots showing the empirical receiver operating characteristic (ROC) curves for acoustic. **(A)** Happy (HA), **(B)** Neutral (NE), **(C)** Fear (FE), and **(D)** Disgust (DI) emotions. Each subplot shows data from all participants. The diagonal broken red line indicates discrimination accuracy at the chance level.

Signal detection theory is advantageous to cognitive psychological experiments wherein two different types of stimuli are to be discriminated against and is widely accepted in the field. For example, SDT is applied to experiments wherein participants discriminate between signals (stimuli) and noise (no stimuli) (Swets and Green, 1978; Stanislaw and Todorov, 1999), such as in our experiment. The above two metrics (Proportion Hits and False alarms) were calculated for each of the six confidence ratings for each target emotion. The six pairs of values were then used to plot the empirical receiver operating characteristic (ROC) curves by linear extrapolation for each Target emotion. Then the empirical area under the ROC curve (eAUC) was estimated as a measure of sensitivity for detecting the Target emotion (signal) not affected by response bias. See Figures 2A–D for an illustration. The empirical ROC curves were estimated by linear extrapolation as it makes no assumptions about the normality of the signal and noise distributions (Stanislaw and Todorov, 1999) and has been used in similar emotion detection experiments earlier (Pessoa et al., 2005; Doty et al., 2013).

**Figure 3:**
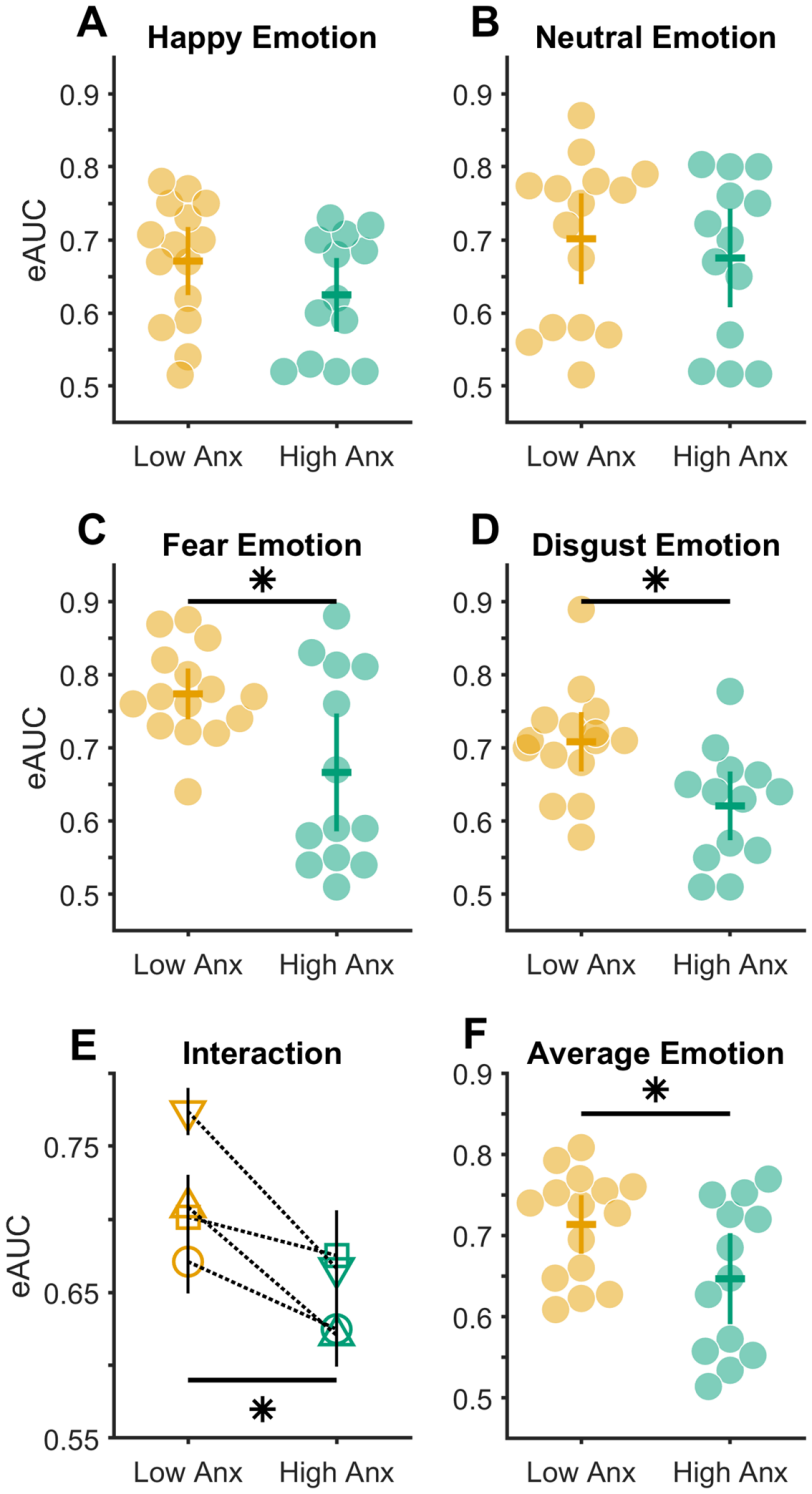
Distribution of empirical AUC values. **(A–D)** Distribution of eAUC values in subsets of participants with low (light orange markers) and high (bluish green markers) anxiety in different emotions. **(E)** Plot showing the interaction between emotions (Happy – circle, Neutral – square, Fear – inverted triangle, Disgust – triangle) and anxiety levels. Solid black vertical error bars show the standard errors of the mean. Note that a few error bars are overlapping. **(F)** Distribution of average eAUC value across all four emotions in subsets of participants with low (light orange markers) and high (bluish green markers) anxiety. The colored horizontal and vertical bars in **(A–D,F)** show means and 95% confidence intervals, respectively, of each distribution; * *p* < 0.05.

The eAUC values reflected the participant’s sensitivity to detecting different Target emotions in the acoustic signal (speech-in-noise). Values of eAUC = 0.50 indicated chance performance, and eAUC values greater than 0.50 indicated relatively higher levels of acoustic emotion detection sensitivity of the participant. This metric (eAUC) was used for all later statistical analyses. Participants reported the experiment to be difficult overall. Two common reasons for this difficulty cited by the participants were background distractions in and around their accommodations and gradually losing focus on the experimental task over the relatively long duration of the experiment (90 min). Thus, only those attaining eAUC values above the chance level (0.50) in all four Target emotions were considered for final analyses to reach the study’s key conclusions (see Results and Discussion). By this criterion, four out of 32 participants who could not cross chance level performance in one or more Target emotions were excluded. Please note that all above 32 participants had passed the hearing acuity test plus the headphone screening tests (details in Participants section) and were subsequently excluded after the complete experiment only for not crossing the aforementioned eAUC criterion of 0.50. The number indicated under the ‘Participants’ section (n = 28) is the final sample size after this exclusion. The average eAUC in all four Target emotions across the included participants (n = 28) was ≥0.65 (range = [0.65, 0.72]). All statistical analyses were carried out with the Statistics and Machine Learning Toolbox of MATLAB 2023a, and the beeswarm plots of Figure 3 were created using the function written on MATLAB as explained here (Stevenson, 2023). For all purposes, two-tailed *p* < 0.050 was considered statistically significant.

## Results

The participant sample (n = 28) had a mean trait-anxiety of 40.11 ± 3.52 (mean ± SD; range = 33–47) and a mean state-anxiety of 34.46 ± 2.74 (mean ± SD; range = 29–42). The sample was then divided into two independent subsets based on the trait-anxiety cut-off score of 39 used earlier (Spielberger, 1983; Kendall and Sheldrick, 2000), such that the subset of participants (*n* = 15) with trait-anxiety scores ≤39 were classified as relatively Low Anxious individuals, and the other subset (*n* = 13) with trait-anxiety scores >39 were classified as High Anxious individuals. The difference of trait-anxiety scores between Low and High Anxious individuals was tested using the Mann Whitney U Test as data in the Low Anxious group violated the assumption of normality (*Lilliefor’s statistic* = 0.22; *p* = 0.009). This showed a lower trait-anxiety in the Low Anxious individuals [median (interquartile range, IQR) = 38 (1.75); range = 33–39] as compared to the High Anxious individuals [median (IQR) = 42 (4.25); range = 40–47], which was statistically significant (*z* = − 4.49, *p* = 6.86 × 10^−6^, *r* = 0.16).

To test whether the levels of anxiety (Low vs. High) influenced the detection sensitivity of different Target emotions, a 2 × 4 MIXED Analysis of Variance (ANOVA) was done with a between-subject factor of anxiety (levels: Low and High) and within-subject factor of Target emotion (levels: HA, NE, FE, and DI). This test when conducted after confirming the adherence of the data to the assumptions of normality (Kolmogorov–Smirnov (K-S) test of normality; all *K-S*_(28)_ statistic ≤0.17, all *p* ≥ 0.05) and homogeneity of variance (Mauchly’s test of sphericity: *Mauchly’s W*_(28)_ = 0.85, *p* = 0.540), returned a significant interaction between the factors of anxiety and Target emotions (*F*_(3,78)_ = 2.776, *p* = 0.047, partial *η^2^* = 0.096; illustrated in Figure 3E). Consequently, this was followed up with simple main effects analyzes. This revealed that the detection sensitivity between Low and High Anxious individuals varied significantly in FE (mean difference ± s.e.m. = 0.107 ± 0.038; Bonferroni corrected *p* = 0.010; Figure 3C) and DI emotion (mean difference ± standard error of the mean, s.e.m. = 0.088 ± 0.029; Bonferroni corrected *p* = 0.005; Figure 3D) respectively. No statistical significance in the detection sensitivity between the Low and High Anxious individuals was found for either the HA (mean difference ± s.e.m. = 0.046 ± 0.032; Bonferroni corrected *p* = 0.157; Figure 3A) or the NE (mean difference ± s.e.m. = 0.026 ± 0.042; Bonferroni corrected *p* = 0.550; Figure 3B) emotions.

Finally, to test whether the overall emotion detection sensitivity varied between Low and High Anxious individuals, the eAUC values across all four emotions were averaged for each participant in both the groups (subsets) of anxiety. Next, as the data in both groups were found to be non-normal in distribution (Kolmogorov–Smirnov (K-S) test, both *K-S _(28)_* statistic ≥0.69, all *p* < 0.05), a Mann–Whitney U test was conducted between the two independent groups. This confirmed a significant difference in emotion detection sensitivity between the Low and High Anxious groups (median difference = 0.09; *z* = 2.005, *p* = 0.045, *r* = 0.556, Figure 3F).

## Discussion

In this online pilot study, we examined whether the sensitivity of detecting vocal emotions in noisy speech (acoustic) signals differed between sub-clinically Low and High Anxious individuals in a less controlled task design wherein the participants discriminated between Target and Non-Target acoustic signals remotely from the natural environment of their accommodations. Consequently, we found that not only did the average detection sensitivities across four different emotions vary between Low and High Anxious individuals, but this effect was particularly conspicuous in the emotions of negative affective valence, i.e., FE and DI. In sum, the findings from our pilot study suggest that a dispositional trait of anxiety in individuals may influence the acoustic appraisal of vocal emotions in noise-ridden, real-world environments and that our study design, with a few considerations, is feasible for a full-scale future research outside of traditional in-laboratory emotion recognition designs.

Existing in-laboratory studies have shown that various aspects of an organism’s internal state, i.e., anxiety and emotional arousal, influence auditory functions in both animals (McGinley et al., 2015) and humans (Pollock et al., 2006; Quadflieg et al., 2007; Koizumi et al., 2011; Pollock-Wurman et al., 2011; Bergman et al., 2016; Peschard et al., 2017; Peschard and Philippot, 2017; Tseng et al., 2017; Zhang et al., 2021). These studies in cognitive (neuro)science have been carried out with tight control of the experimental variables such that the variable of investigation/interest is isolated, holding all others constant. This is generally done to screen out irrelevant confounding variables for discovering the latent brain-behavior relationships (if any) in the variable(s) of interest (Nastase et al., 2020). While such studies are of value, and their findings stand a good chance of being reproduced in precisely the same experimental design in another instance, a high degree of experimental control may compromise the generalizability of these findings to more naturalistic contexts. To address this particular aspect, it has been argued that existing study designs in cognitive (neuro)science be complemented with designs that are more ecologically valid that better capture the brain-behavior relationships in natural environments with several uncontrolled variables within which our brains have evolved to guide behavior (Nastase et al., 2020). Our study design takes a step in this direction by losing control over the participants’ ambient environment and measuring participants’ responses to the acoustic stimuli remotely through an online platform. Consequently, our findings that trait-anxiety influences the detection sensitivity of vocal emotions corrupted by background noise attests to the earlier in-laboratory evidence of a link between anxiety and emotional arousal in auditory perception (Pollock et al., 2006; Quadflieg et al., 2007; Koizumi et al., 2011; Pollock-Wurman et al., 2011; Bergman et al., 2016; Peschard et al., 2017; Peschard and Philippot, 2017; Tseng et al., 2017; Zhang et al., 2021), but only extend the results beyond a controlled laboratory environment. While online experiments in cognitive science have been popular for some time now, experiments in the domain of auditory psychophysics have been relatively scant, partly owing to the challenges of presenting acoustic signals on personal computer speakers with fidelity (Woods et al., 2017; Milne et al., 2021). We, through several steps in our pilot design, show the possibility of addressing those challenges in answering questions of auditory emotion recognition from experiments with more extensive and diverse samples online.

Anxiety is an apprehension about an impending threat without any sound rationale and is characterized by nervousness, worry, and activation of the autonomic nervous system (arousal). Trait-anxiety is a relatively stable predisposition (personality trait) indexed by the trait subscale of the STAI. By contrast, the state subscale of the STAI indexes the characteristics mentioned above of subjective anxiety at the given moment of administering the instrument and is better reflected in experimental paradigms with a direct anxiety induction step (Spielberger, 1983, 2022; Wiedemann, 2015). Several earlier studies, particularly in the visual domain, have reported biases in sensory processing on account of anxiety and, more importantly, point to a close link between anxiety and processing of environmental affect, e.g., emotions (Bishop, 2007, 2009; Bocanegra and Zeelenberg, 2011; Ferneyhough et al., 2013; Mogg and Bradley, 2016; Barbot and Carrasco, 2018; Chakrabarty et al., 2021; Kaur et al., 2023). Along these lines, reports of a clear association between anxiety and auditory affective processing, e.g., detection of emotions from acoustic signals in humans, have been scarce in the literature, to our knowledge. Our study here fills in this gap and outlines one way for the exploration of our preliminary findings in larger and more diverse samples. In measuring the differences of an objectively quantifiable aspect of auditory affect, we have grouped the otherwise subclinical individuals into Low and High Anxiety based on scores reflecting the dimensional representation of trait-anxiety on a continuum, e.g., STAI. Nevertheless, we think that our findings may extend our understanding of dysregulated auditory behavior in other clinically diagnosed affective disorders as well since the extant literature suggests anomalous auditory processing of varying natures with distinct neural correlates in individuals with autism (Čeponienė et al., 2003), schizophrenia (Gold et al., 2012; Javitt and Sweet, 2015; Conde et al., 2016) and depression (Zweerings et al., 2019; Bissonnette et al., 2020).

Auditory perception of speech proceeds through several feature extraction steps at different stages of neural processing, involving a dynamic cross-talk between bottom-up (sensory-driven) and top-down (experience/context-driven) processes in the brain (Schroeder et al., 2010). There have been reports of the contribution of prior experience/context-driven processes in facilitating auditory neurons in filtering out noise from task-relevant acoustic signals at any given moment, toward processing auditory information (Giraud and Poeppel, 2012; Fontolan et al., 2014; Holdgraf et al., 2016). It is thus plausible, that in our experiment, the subjective behavioral context (e.g., trait-anxiety, emotional arousal) was also a critical factor in modulating the efficiency of filtering negative affective speech signals (FE and DI) out of white background noise, thereby influencing the auditory sensory gain and ultimately auditory emotion perception as reflected by the detection sensitivity in our results. The fact that the effects in our data were observed with predominantly negative, threatening affective signals (FE and DI) could be explained on the premise that such environmental stimuli are processed faster and with more efficiency in the brain towards influencing behavior (Davidson et al., 2004). These conclusions from our results relate to the findings of a recent study that also points to different induced moods influencing masked auditory detection thresholds in humans. However, somehow, their data did not allow for drawing unequivocal conclusions (Bolders et al., 2017). Our findings of lower auditory emotion detection sensitivity with higher anxiety are in contrast to earlier reports of enhancements in visual sensitivity with greater anxiety, especially for negative stimuli (Byrne and Eysenck, 1995; Bradley et al., 1998; Fox, 2002; Richards et al., 2002; Koster et al., 2006b; Bar-Haim et al., 2007; Doty et al., 2013). These incongruent findings between two different sensory domains (auditory vs. visual) may be due to a few reasons. One, the lower detection rates of negatively arousing FE and DI emotions from speech could be related to differential recognisability of acoustic emotions as reported earlier (Banse and Scherer, 1996; Wildgruber et al., 2005), which was pronounced in our sample with higher trait-anxiety. Two, it is also possible that the effect higher trait-anxiety has on the acoustic detection sensitivity of certain vocal emotions, e.g., those conveying negative affect and especially, requiring more intensive and longer cerebral processing as in DI (Wildgruber et al., 2005; Pell and Kotz, 2011), varies from visual sensory domain. This particular aspect merits future enquiry.

Finally, we identify a few issues of our study which may be considered for interpreting the results and for addressing them in future studies. First, since this was a pilot study with lesser resources, we tried to maximize the yield from our small sample size by increasing the number of trials (total 240), focussed on each experimental condition (60 trials for emotion valence) as discussed here (Hopkin et al., 2015). However, we think that the findings from our pilot study will gain more credence if the results could be replicated in a full-scale study with a larger sample. Second, there was some attrition in our analyzed sample (four out of 32, ~ 12.5%) as their experimental task performance did not exceed chance level detection sensitivity. Based on participant feedback, a few ways of addressing this could be to reduce the entire task duration by a) using shorter speech clips (< 3,000 ms), b) reducing the intervening blank duration per trial proportionately (< 3,000 ms), and c) exploring fewer emotions in an experiment to reduce the total task duration without compromising on the number of trials per emotion. Third, the trait-anxiety subscale of STAI overlaps with measures of depression (Caci et al., 2003), which we cannot rule out from our results. Thus, our results may apply generally be interpreted as a trait of negative affectivity rather than specifically of the trait of anxiety, which may be addressed in future studies by including a measure of depression also, such as explained here (Kroenke et al., 2001). Fourth, pre-screening larger participant samples in a full-fledged study and recruiting participants into Low and High-trait anxiety groups based on their STAI scores as explained earlier (Koster et al., 2006a) would be better for clarifying the pattern of emotion detection sensitivities between the two independent groups with greater differences (compared to our present samples) of trait-anxiety between them.

In conclusion, our preliminary results demonstrate that the detection sensitivity of temporally unpredictable affect from human speech embedded in background white noise is compromised in relatively high as compared to low levels of trait-anxiety in subclinical individuals. This effect is particularly pronounced in speech, conveying negative and threat-related affect. These findings supplement the literature on the interplay of affective states and information on auditory processing. The experimental design and its findings may also help in designing similar, larger-scale, workable study protocols to aid a better understanding of the auditory processing deficits in affective states and disorders.

## Data availability statement

The raw data supporting the conclusions of this article will be made available by the authors, without undue reservation.

## Ethics statement

The studies involving humans were approved by Institutional Review Board, Indraprastha Institute of Information Technology Delhi, India. The studies were conducted in accordance with the local legislation and institutional requirements. The participants provided their written informed consent to participate in this study.

## Author contributions

AK: conceptualization, methodology, investigation, software, and writing. SP: methodology, investigation, and data curation. MC: funding acquisition, conceptualization, methodology, investigation, software, formal analysis, writing – original draft preparation and revision, supervision, and project administration. All authors contributed to the article and approved the submitted version.

## Funding

The research was funded by the Research Initiation Grant by IIIT-D as well as by a grant from the Center for Design and New Media (A TCS Foundation Initiative supported by the Tata Consultancy Services) at IIIT-Delhi to Dr. Mrinmoy Chakrabarty. The funders were not involved in the study design, collection, analysis, interpretation of data, the writing of this article or the decision to submit it for publication.

## Conflict of interest

The authors declare that the research was conducted in the absence of any commercial or financial relationships that could be construed as a potential conflict of interest.

## Publisher’s note

All claims expressed in this article are solely those of the authors and do not necessarily represent those of their affiliated organizations, or those of the publisher, the editors and the reviewers. Any product that may be evaluated in this article, or claim that may be made by its manufacturer, is not guaranteed or endorsed by the publisher.
